# Genomic Regions Associated With Interspecies Communication in Dogs Contain Genes Related to Human Social Disorders

**DOI:** 10.1038/srep33439

**Published:** 2016-09-29

**Authors:** Mia E. Persson, Dominic Wright, Lina S. V. Roth, Petros Batakis, Per Jensen

**Affiliations:** 1 AVIAN Behaviour Genomics and Physiology Group, IFM Biology, Linköping University 581 83, Linköping, Sweden

## Abstract

Unlike their wolf ancestors, dogs have unique social skills for communicating and cooperating with humans. Previously, significant heritabilities for human-directed social behaviors have been found in laboratory beagles. Here, a Genome-Wide Association Study identified two genomic regions associated with dog’s human-directed social behaviors. We recorded the propensity of laboratory beagles, bred, kept and handled under standardized conditions, to initiate physical interactions with a human during an unsolvable problem-task, and 190 individuals were genotyped with an HD Canine SNP-chip. One genetic marker on chromosome 26 within the *SEZ6L* gene was significantly associated with time spent close to, and in physical contact with, the human. Two suggestive markers on chromosome 26, located within the *ARVCF* gene, were also associated with human contact seeking. Strikingly, four additional genes present in the same linkage blocks affect social abilities in humans, e.g., *SEZ6L* has been associated with autism and *COMT* affects aggression in adolescents with ADHD. This is, to our knowledge, the first genome-wide study presenting candidate genomic regions for dog sociability and inter-species communication. These results advance our understanding of dog domestication and raise the use of the dog as a novel model system for human social disorders.

Dogs are unique among domesticated animals, having been integral parts of human societies and families for at least 15 000 years^1^. During the course of domestication they have evolved several social skills enabling them to communicate and cooperate with humans to an extent unmatched by any other species^2^,^3^. For example, they form strong attachment bonds with their owners^4^, are sensitive to different types of human ostensive cues and are able to comprehend referential gestures as well as emotional facial expressions^5^,^6^,^7^.

Dogs also show intentional referential communicative behavior towards humans, involving both attention-seeking and directional components e.g. in problem-solving situations^8^,^9^,^10^. Different lines of evidence suggest a substantial genetic basis for these social skills. For example, even young puppies are able to read human communicative signals while socialised wolves largely lack the ability^11^. Unlike wolves, dogs usually turn to a nearby human in a help-seeking manner when faced with difficult problem tasks^12^,^13^,^14^. We recently reported significant heritabilities for such communicative behavior in a population of laboratory beagles^10^. In the present study, we used this population to perform a genome-wide association study (GWAS) in order to map candidate genes associated with the social skills of the dogs. The dogs were all raised under highly standardised conditions with similar experiences of human contact, which makes them particularly suitable for exploring the genetic basis of the behavior.

The genetic basis for variation in social behavior is crucial to understand in order to unravel its evolution and domestication. The “nature-vs-nurture” of social behavior has been studied in humans and other mammals, as well as in social insects^15^. However, previous studies generally focus on interactions between members of the same species, while genetics of interspecies communication in mammals – a central aspect of domestication – have received less attention. For the purpose of studying communication between species, the dog, with its well-developed human-directed affability, is a powerful model. Previous research has focused on a few candidate genes, such as the oxytocin-receptor gene^16^, but no systematic search for genes has so far been performed.

On the other hand, dogs are widely recognized as being particularly suitable models for disease genetics, due to its favorably structured genome^17^,^18^. Lindblad-Toh, *et al*.^18^ suggests that 15.000 evenly spaced informative SNPs should be sufficient for association studies on disease alleles for simple Mendelian dominant traits. Here, we rather use a within-breed association study to search for genetic associations with quantitative traits (social behavior), which has the advantage of reducing locus heterogeneity, similar to what is seen in studies of geographically isolated human populations in countries such as Iceland or Finland^19^.

In the current study, we conducted a dense genome-wide association study (GWAS) on human-directed social behaviors in a population of beagles bred, kept and handled under highly standardized conditions. The aim was to identify possible candidate genes, which can increase our understanding of the evolution of domesticated social behavior in dogs.

## Results

We studied dogs when they were individually presented with an unsolvable problem. The dogs, all genotyped with an HD Canine SNP-chip, were allowed to manipulate a device where they could easily obtain two treats, but a third had been made inaccessible. This caused most of the dogs, at some point, to turn to the nearby human and seek cooperation by gazing towards the eye region and through physical proximity and contact. We recorded the propensity of each dog for searching such human contact and found three SNP-associations, one significant and two suggestive, situated within two different genes on chromosome 26. Three other candidate genes were present in one of the linkage blocks. Interestingly, four of the five genes thus identified have previously been associated with social behavior disorders in humans, and therefore remain strong candidates for modifying human-directed behavior in dogs. The details of our results are given below.

The chip heritability estimates of the different human-directed social behaviors ranged from 0 for latency to enter human proximity, to 0.37 ± 0.16 (SE) for duration spent in human proximity (Table 1).

**Figure 1:**
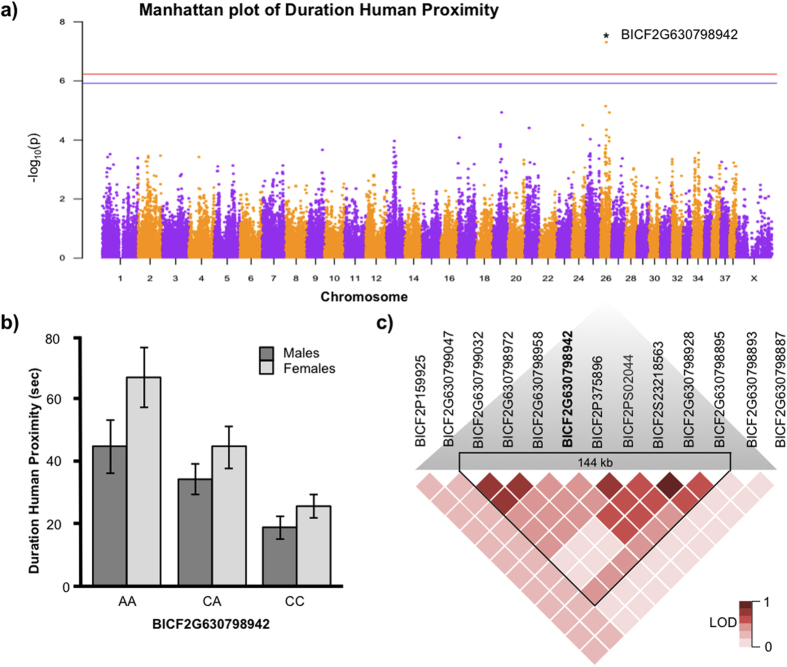
Results from Genome-Wide Association analysis on the Human Proximity behavior. (**a**) Manhattan plot where * indicates the significant SNP on chromosome 26. Significance levels are shown by the red line (Bonferroni 5%) and the blue line (Bonferroni 10%). (**b**) Histogram showing the behavioral phenotype of each genotype for the significant SNP, separated between males and females. Error bars are ± 1 SE. (**c**) Linkage disequilibrium heat map of the SNPs surrounding the significant SNP.

**Figure 2:**
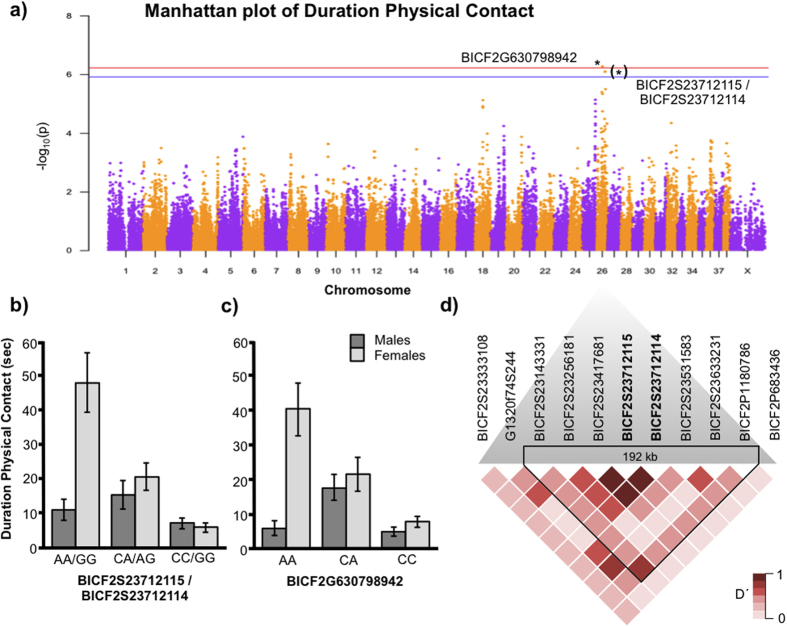
Results from Genome-Wide Association analysis on the Physical Contact behavior. (**a**) Manhattan plot where * indicates the significant SNP on chromosome 26, (*) indicated the suggestive SNPs also on chromosome 26. Significance levels are shown by the red line (Bonferroni 5%) and the blue line (Bonferroni 10%). (**b**) Histogram showing the behavioral phenotype of each genotype for the suggestive SNPs, separated between males and females. Error bars are ± 1 SE. (**c**) Histogram showing the behavioral phenotype of each genotype for the significant SNP, separated between males and females. Error bars are ± 1 SE. (**d**) Linkage disequilibrium heat map of the SNPs surrounding the suggestive SNPs. See Fig. 2. (**d**) for the linkage disequilibrium heat map for the significant SNP.

We found one significant and two suggestive SNP-associations with human-directed social behaviors (Table 2, Figs 1a and 2a). The SNP BICF2G630798942 on chromosome 26, situated within intron 9 out of the 16 interspersed introns of the *SEZ6L* gene (Ensemble 81, ENSCAFG00000011694.3), was significantly associated with the time dogs spent in close proximity of the human as well as in direct physical contact (Fig. 1a). Both sex (Univariate GLM: F_1,186_ = 5.146, p = 0.02) and genotype (Univariate GLM: F_2,186_ = 16.101, p < 0.001) had significant effects where females had higher behavior scores than males across all genotypes (Fig. 1b). More time spent in close proximity to humans was found in dogs possessing the ‘A’ allele in both males and females at this locus, while heterozygotes (‘CA’ alleles) had intermediate values. No other genes are present within the 144 kb linkage disequilibrium window (Fig. 1c). The haplotype containing the significant SNP and its closest most associated neighboring SNP, had a significant effect on the “human proximity” phenotype (Univariate GLM: F_6,176_ = 4.820, p < 0.001)(Supplementary Figure S1). No 2-SNP haplotype effect was found for the “physical contact” phenotype.

Furthermore, two other closely linked SNPs on chromosome 26, (BICF2S23712115 and BICF2S23712114), were suggestively associated with the duration of seeking human physical contact (just below the threshold for significance) (Fig. 2a). These two SNPs are located within intron 1 of the 18 introns of the *ARVCF* gene (Ensembl 81, ENSCAFG00000014232). In females, again the ‘A’ allele was associated with higher score for physical contact, with the heterozygotes (‘CA’) showing intermediate scores (Fig. 2b). In males, on the other hand, the heterozygotes had the highest scores, possibly suggesting an over-dominance effect. Within the 192 kb linkage disequilibrium window surrounding these SNPs (Fig. 2c), there are three additional genes present (*TXNRD2*, *COMT* and *TANGO2*).

Allele frequencies and phenotype means for the significant and suggestive SNPs are presented in Supplementary Table S2. Table 2 presents p-values for significant (p < 5.9E-7; Bonferroni p < 0.05) and suggestive (p < 1.2E-6; Bonferroni p < 0.1) SNPs. Effect sizes (R-squared) and related genes are presented in Table 3. No significant or suggestive behavior-SNP associations were found for the remaining behavior phenotypes.

In order to validate the CanineHD BeadChip genotyping, 30 individuals were also genotyped for the BICF2G630798942 and BICF2S23712114 SNPs with pyrosequencing resulting in 100% accordance. Furthermore, an additional sample of 69/70 individuals from the phenotyped population was genotyped for these SNPs and added to the 190 dogs from the GWAS generating very similar results as previously shown in Figs 1b and 2b,c. The results from the analysis with 190 + 69/70 individuals are presented in Supplementary Figures S3–S5. Also, the results from the analysis of the 69/70 individuals are presented in Supplementary Figures S6–S8.

## Discussion

This study used a high-density canine SNP-chip to genotype a unique population of laboratory beagles tested in the unsolvable problem paradigm. This problem stimulates dogs to seek human attention and display human-directed contact seeking behaviors. Through a GWAS, we identified genomic regions containing five possible candidate genes, associated with human-directed social behaviors. To our knowledge, this is the first study reporting strong evidence for specific genes associated with social skills known to have developed during the domestication of dogs.

Wilsson and Sundgren^20^ found heritability estimates very similar to ours (0.42 ± 0.10 (SE)) for social contact when testing 8-week-old German Shepherd puppies. Furthermore, van der Waaij, *et al*.^21^ found comparable heritability for a subjective score of dogs’ willingness to seek human contact (affability) for German Shepherd Dogs (0.38 ± 0.06 (SE)) but not in Labrador Retrievers (0.03 ± 0.03 (SE)). This corroborates our findings and suggests a clear genetic contribution to variations in human-directed social behavior in dogs.

Even though we cannot with certainty establish any linkage with specific genes, the genomic regions identified contain highly interesting genes with known association to human social disorders. The association study revealed a significant association with the *SEZ6L* gene in our study population, and showed that no other gene was present within the linkage disequilibrium window surrounding the SNP. Additionally, analysis of the haplotype of the SEZ6L-SNP and its closest neighboring SNPs showed that the main effect derives from the significant SNP. In humans, *SEZ6L* has recently been linked with autism spectrum disorder, which is a neurodevelopmental disorder connected with impairment in social interactions and communication^22^. A genome-wide study on American families with autistic children found a significant relation between the genotype at a SNP-marker close to this gene and the risk of being diagnosed with autism. Previously, polymorphisms in *SEZ6L* have also been associated with bipolar disorder including a gene x sex interaction, affecting susceptibility in women but not in men^23^, once again mirroring the results observed in our study animals with this polymorphism.

Two further suggestive associations were found with *ARVCF*, which in humans has previously been associated with schizophrenia^24^,^25^,^26^,^27^. Three more genes were found within the same linkage block and out of those, *TXNRD2* and *COMT* have previously been associated with schizophrenia and social disorders^28^. *COMT* is involved in catabolizing cathecholamines such as dopamine and a common polymorphism, the Val158Met, is associated with mood regulation and contributes to variation in aggression in adolescents with attention-deficit hyperactivity disorder (ADHD)^29^,^30^. This polymorphism has also been linked to the “persistence” personality trait in humans^31^ as well as the risk of developing schizophrenia^32^. Interestingly, the same *COMT*-polymorphism is also present in dogs, and its frequencies vary between breeds, suggesting that it could play an important role in regulating behaviors^33^.

An additional sample of 69/70 dogs from the original population was genotyped for both the significant and the suggestive SNP. Adding these results to the previously genotyped 190 individuals did not change the results significantly. On the other hand, no effects of sex or genotype were found within the additional sample on its own. These findings are not surprising as the additional individuals all belong to the intermediate phenotype group as the top 95 and tail 95 dogs were chosen for the initial analysis, hence reducing the power to detect genotypic effects. However, there is a possibility that the results found in the full dataset are false positives, potentially related to the small sample size. In order to validate the current results, additional independent populations of laboratory beagles raised under similar conditions can be analyzed in a replicating study. Another possibility would be to investigate the effects of these genotypes on human-directed social behaviors in several populations of different breeds.

In previous research, high prevalence of breed specific diseases such as heart disease, cancer, blindness, epilepsy and psychological disorders such as obsessive compulsory disorder (OCD), have raised the interest of dogs as models of human disorders^34^. For example, genes associated with epilepsy^35^ and narcolepsy^36^ were first mapped in dogs. Although our study is mainly concerned with finding genes related to behavior traits, which presumably have been under strong selection during domestication, it also illustrates the potential of the dog as a human behavior model. It is striking that the genes we identified are involved in human social behavior disorders. Hence dogs may be highly valuable for mapping behaviors and behavioral disorders specific to both the dog as a species^37^ and to particular breeds^38^, as well as human psychological disorders such as anxiety, OCD and autism^39^. For example, OCD is phenotypically very similar in dogs and humans and candidate genes were first mapped in Doberman pinchers^40^.

In conclusion, this is to our knowledge the first GWA study to present genomic regions containing putative candidate genes associated with interspecies communication in dogs, presumably under strong selection pressures during domestication. The identified genes have previously been associated with human social impairments, such as autism. These results contribute to a greater insight into the genetic basis of dog-human communicative behaviors and sociability, increasing our understanding of the domestication process, and could potentially aid knowledge relating to human social behavioral disorders.

## Materials and Methods

### Animals and behavior phenotyping

The genotyped dog sample consisted of 190 laboratory beagles ranging between 6.2 and 0.8 years, with an average of 1.8 years, at the time of testing. These individuals were selected from a total of 437 dogs previously tested as described below. The behavioral test results rendered four principal components, of which the second was associated with human-directed behavior, showing significant heritability^10^. We therefore selected dogs with the 95 top and 95 tail scores on the second principal component for the present analysis. The dogs were bred, kept and handled under highly standardized conditions at a research dog kennel in the south of Sweden. Although the population was intentionally outbred, many of the dogs were related on different levels and some degree of inbreeding occurred, which has to be taken into consideration in the analysis. The behavioral study of these dogs has been published previously and more detailed information on handling, housing, ethograms and testing can be found there^10^.

Briefly, dogs were each tested once using an unsolvable task paradigm consisting of three identical problems of which one was unsolvable. The task was to slide a Plexiglas lid to the side in order to obtain a treat in a container underneath. One of the three lids had been fixed and could not be opened, which constituted the unsolvable part of the problem. In the procedure room a female researcher, previously unknown to the dogs, was facing the test setup sitting passively on a stool approximately 1.5 meters from the problem-solving device, which was placed on the floor, while the dogs were left to move freely in the room for 3 minutes. The behavior tests were video recorded and later analyzed using the Noldus Observer X 10 software. Prior to testing, dogs’ willingness to eat the presented treats had been scored and dogs that did not eat the treats were excluded from the study.

The human-directed social behaviors analysed in the current study are presented in the ethogram (Table 4) and includes physical contact and staying in close proximity to the human. In the previous study, behaviors were summarized through a principal component analysis (PCA) resulting in 4 principal components (PCs) named test interactions, social interactions, eye contact and physical contact based on the behaviors with the highest and lowest scores within each PC^10^. Statistical analysis showed that sex had a significant effect on the PC related to social interactions where females scored higher than males (Univariate GLM; F_1,432_ = 17.60; p < 0.01). Effects of sex and age on each of the behaviors used in this study can be found in ref. 10. Additionally, narrow-sense heritability (h^2^) was estimated to 0.23 (HPD: 0.06–0.42) for the PC related to social interactions using MCMCglmm in R.

In the present study, GWAS was performed as described below on the behaviors listed in Table 4 as well as on the scores on the second principal component (PC2) from Persson, *et al*.^10^ related to social interactions and the third principal component (PC3) related to eye contact-seeking behaviors.

### DNA sampling, extraction and genotyping

All experimental protocols were approved by the Swedish ethics committee in Lund, Sweden (21 June 2012), and the methods were carried out in accordance with the relevant guidelines. DNA was obtained either from buccal cells or from blood samples. Buccal cell DNA was collected with and extracted from buccal swabs using the standard protocol of the Isohelix kit DDK-50. Samples were kept in Lysis Buffer and proteinase K for 48 hours prior to continuing with the protocol. Single 50 μl elutions were used. DNA was extracted from whole blood samples using the standard protocol of the QIAGEN QIAamp DNA mini kit (DNA Purification from Blood or Body Fluids, Spin Protocol). Single 100 μl elusions were used. Subsequently, DNA was quantified using a Nanodrop ND-1000 and stored at −20 °C until further use. DNA samples from 95 dogs with the top PC2 scores and 95 dogs with the lowest PC2 scores (in total 190 samples filling two plates) were genotyped with the Illumina’s Infinium assay and the CanineHD BeadChip at the SNP&SEQ Technology Platform, Uppsala University^41^,^42^.

### Quality control

Initial quality control was performed at the SNP&SEQ Technology Platform in Uppsala, using the software GenomeStudio 2011.1 from Illumina Inc. This was done in order to confirm both sample and marker quality^43^. All individuals had call rates >98%, which was above the 90% threshold, hence no samples were removed.

Further quality control was performed on 172 115 SNP markers using the PLINK 1.07 analysis software (http://pngu.mgh.harvard.edu/~purcell/plink/)^44^. Markers failing the criteria of Hardy-Weinberg expectations p > 0.05, genotyping rate > 90% and minor allele frequency (MAF) of >5% were removed finally leaving 85 172 markers for the association analysis.

### Genome-wide association study

Raw phenotype data can be found in Supplementary Data S9 and genotype data in Supplementary Data S10 & S11 publicly available at https://osf.io/m5a3v/?view_only=a6e3a4383f894d7cbda34c0750f26fb0. PLINK v1.07 was used to perform the initial naïve genome-wide association analyses (GWAS)^44^. Genome-wide corrected empirical p-values were determined by 100.000 max (T) permutations. The genomic inflation factor (λ) was ranging from 1.0–1.4 for the different phenotypes suggesting a minor level of population stratification. To adjust for population stratification and relatedness the rest of the GWAS was performed using the GEMMA software (http://www.xzlab.org/software.html). GEMMA implements the Genome-wide Efficient Mixed Model Association algorithm for GWAS^45^. First, a centered relatedness matrix was estimated from genotypes for each phenotype. The association tests were then performed with the relatedness matrix, phenotype, genotype and the fixed effects (sex and/or age) fit into a univariate linear mixed model as follows:


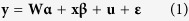


where **y** is a vector of phenotypes, **W** is a covariate matrix with the vector **α** of the fixed effects including the intercept, **x** is a vector of marker genotypes and the effect size of the marker is **β**, **u** is a vector of random effects and **ε** is a vector of residual errors. Additionally, GEMMA estimates the proportion of variance in the phenotypes explained (PVE or “chip heritability”). The Wald frequentist test was chosen to test for significance in GEMMA and to determine the genome-wide significance the Bonferroni threshold for p < 0.05 for 85 172 tests was calculated. In order to make sure that the population stratification had been accounted for, GenABEL in R was used on the Wald p-values to make a new estimate of λ using the regression method^46^. To generate Manhattan and Q-Q plots the qqman R-package was used. Q-Q plots of GEMMA p-value distributions for each of the studied behaviors can be found in Supplementary Appendix S12. The genomic inflation factor (λ) for each behavioral phenotype, before and after accounting for population structure, is presented in Table 1 together with PVE or “chip heritability” estimates. We studied a laboratory beagle population, which had been intentionally outbred, but there was still some population stratification present in the tested individuals that can be seen in the naïve (raw) genomic inflation factor (λ) in Table 1. Conveniently, the GEMMA software is able to account for population structure as well as inbreeding and covariates, which is demonstrated by the λ-value being much closer to 1.0 in the GEMMA analysis. This is further supported by the Q-Q plots of the GEMMA Wald p-values in Supplementary Appendix S12. To visualize the population structure, a multidimensional scaling (MDS) analysis was performed in PLINK and the first and second dimensions were plotted in R with top and tail dogs indicated (Supplementary Figure S13).

Behavioral means for the different genotypes of the significant and suggestive SNPs were analyzed through univariate general linear models in IBM SPSS Statistics 22. Subsequent linkage disequilibrium (LD) analysis and visualization was done using the Haploview v4.2 software^47^. LD windows were estimated based upon visual examination of the combined D’ and LOD heat maps. SNPs that were considered to be in LD had D’ of 1 and LOD-score between 0.15 and 1. Additionally, the effect of the haplotype consisting of the significant SNP and its closest neighboring SNP, with the highest association, was tested using univariate general linear models.

### Validation and genotyping through pyrosequencing

In order to validate the GWAS results, 70 samples from the original population of 500 beagles were genotyped for the BICF2G630798942 and BICF2S23712114 SNP markers using polymerase chain reaction (PCR) and subsequent pyrosequencing. Additionally, another 30 samples previously genotyped with the CanineHD BeadChip were analyzed to validate the genotyping. Briefly, primers were designed using the software PyroMark Assay Design 2.0.1.15 by QUIAGEN©. The primers used for BICF2G630798942 were: forward biotinylated in 5′ CTGCCAGGGACTCCTGAG, reverse CTCAAGGCAGCCCATCACT and sequencing reverse GGAGGCTTGCTGCCG. For the BICF2S23712114 SNP the primers used were: forward biotinylated in 5′ CATGTCACAGTTGAGGGGATAGGT, reverse TCTTCAGACAGCCCACCCA and sequencing reverse CAGTCCAGGAAGGAATA. The PCR-mixture contained, for each sample, 0.12 μl DreamTaq™ DNA Polymerase 5 u/ μl (Thermo Scientific), 2,5 μl of 10X DreamTaq™ Buffer (Thermo Scientific), 0.5 μl dNTP 10 mM (2.5 mM each, BIOLINE), 0.5 μl of each primer diluted to 5 μM (Invitrogen), 19.9 μl of nuclease free water and approximately 100–200 ng of DNA template. The final PCR volume was 25 μl for each sample and the reaction was run on the Palmcycler PCR by Corbett. The PCR cycle consisted of an initial denaturation at 95° for 3 min, 40 cycles of 30 s denaturation at 95°, 30 s annealing at 63° for the BICF2G630798942 primers and 61° for the BICF2S23712114 primers, 30 s extension at 72° and a 10 min final extension at 72°.

Pyrosequencing was performed on the entire PCR product according to the PyroMark Q24 Vacuum Workstation Quick-Start Guide found at www.qiagen.com. The results were analyzed using the PyroMark Q24 2.0.6 software. For the BICF2G630798942 SNP, one sample had to be excluded due to PCR amplification failure resulting in a total of 69 additional individuals. The genotypes of the 70 additional individuals were analyzed together with the previous 190 individuals using Univariate GLM in SPSS Statistics.

## Additional Information

**How to cite this article**: Persson, M. E. *et al*. Genomic Regions Associated With Interspecies Communication in Dogs Contain Genes Related to Human Social Disorders. *Sci. Rep.* **6**, 33439; doi: 10.1038/srep33439 (2016).

## Supplementary Material

Supplementary Information

Supplementary Table S2

Supplementary Dataset S9

## Figures and Tables

**Table 1:** Genomic inflation factor (λ) from the naïve GWAS in PLINK (λ_raw_) and the corrected model in GEMMA (λ_GEMMA_).

Phenotype	λ_raw_	λ_GEMMA_	PVE	SE (PVE)
Social interactions (PC2)	1.25	1.02	0.14	0.13
Duration Human Proximity	1.38	1.03	0.37	0.16
Latency Human Proximity	1	0.8	0	—
Frequency Human Proximity	1.11	1.05	0.05	0.09
Duration Physical Contact	1.25	1.08	0.10	0.11
Latency Physical Contact	1.43	1.04	0.13	0.12
Frequency Physical Contact	1.27	1.04	0.14	0.13

PVE or “chip heritability” estimates from the GEMMA analysis are also presented.

**Table 2:** P-values for significant and suggestive SNPs from each behavioral phenotype.

SNP	P_genome_ Max (T)	P_raw_	Beta P_raw_	SE (Beta P_raw_)	P_GEMMA_	Beta P_GEMMA_	SE (Beta P_GEMMA_)
**Duration Human Proximity**							
BICF2G630798942	1.00e-05	2.03e-08	−18.40	3.14	4.87e-08	−17.73	3.12
**Duration Physical Contact**							
BICF2G630798942	1.00e-05	2.23e-07	−11.74	2.18	5.25e-07	−11.25	2.16
BICF2S23712115	1.00e-05	1.31e-07	−12.89	2.35	7.94e-07	−12.07	2.36
BICF2S23712114	1.00e-05	1.31e-07	−12.89	2.35	7.94e-07	−12.07	2.36

**Table 3:** R-squared and position within the genes for the significant and suggestive SNPs from each behavioral phenotype.

SNP	Chr	Location (bp)*	R^2^ SNP	R^2^ SNP & Covariate†	Gene	Position
**Duration Human Proximity**						
BICF2G630798942	26	20025266	0.156	0.178	SEZ6L	Intron 9/16
**Duration Physical Contact**						
BICF2G630798942	26	20025266	0.134	0.220	SEZ6L	Intron 9/16
BICF2S23712115	26	29319347	0.139	0.219	ARVCF	Intron 1/18
BICF2S23712114	26	29319675	0.139	0.219	ARVCF	Intron 1/18

^*^CanFam 3.1.

^†^sex as covariate.

**Table 4:** Ethogram of the human-directed social behavior phenotypes used.

Behavior	Type of measurements	Description
Human proximity	Duration	Total time (s) the dogs’ head is within its body length from the researcher.
Human proximity	Latency	Initial time until the dogs’ head is within its body length from the researcher.
Human proximity	Frequency	Total amount of times the dogs’ head is within its body length from the researcher.
Physical contact	Duration	Total time (s) that the dog is positioned at the researcher and in physical contact.
Physical contact	Latency	Initial time until the dog is positioned at the researcher and in physical contact.
Physical contact	Frequency	Total amount of times he dog is positioned at the researcher and in physical contact.
