# Supplementary material for: Genomic Regions Associated With Interspecies Communication in Dogs Contain Genes Related to Human Social Disorders
Source: Sci Rep. 2016 Sep 29;6:33439. doi: 10.1038/srep33439 (PMC5041581; doi:10.1038/srep33439)
Supplement: Supplementary Information [file srep33439-s1.pdf]

Supplementary S1, S3-S8, S12-S13

Genomic regions associated with interspecies communication in dogs contain genes related to human social disorders

Mia E. Persson[a], Dominic Wright[b], Lina S.V. Roth[c], Petros Batakis[d] and Per Jensen[e]*

Supplementary Figure S1

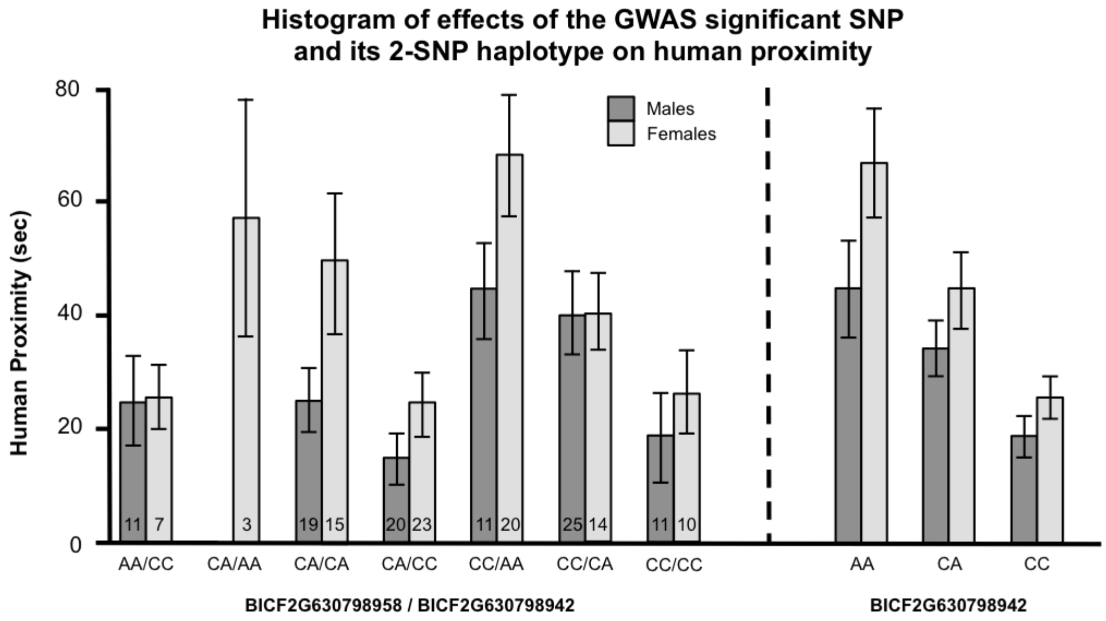

Figure S1: Histogram showing the mean of the behavior "human proximity" of each haplotype of the BICF2G630798942 SNP and its closes neighboring SNP BICF2G630798958, in a population of 190 beagles. Only one individual had the AA/CA haplotype and was excluded from the analysis. The data is separated into males and females, numbers within the bars indicate the number of individuals representing that bar and error bars are +/- 1 SE. Both haplotype (Univariate GLM: $F_{6, 176} = 4.820$, $p < 0.000$) and sex (Univariate GLM: $F_{1, 176} = 4.849$, $p = 0.03$) had significant effects, $R^2 = 0.21$. Comparing the pattern of the different haplotypes with the graph to the right, it looks like the number of A-alleles in the BICF2G630798942 SNP has the greatest impact on the phenotype.

Supplementary Figure S3 – S8

BICF2G630798942 genotype effects on Human Proximity

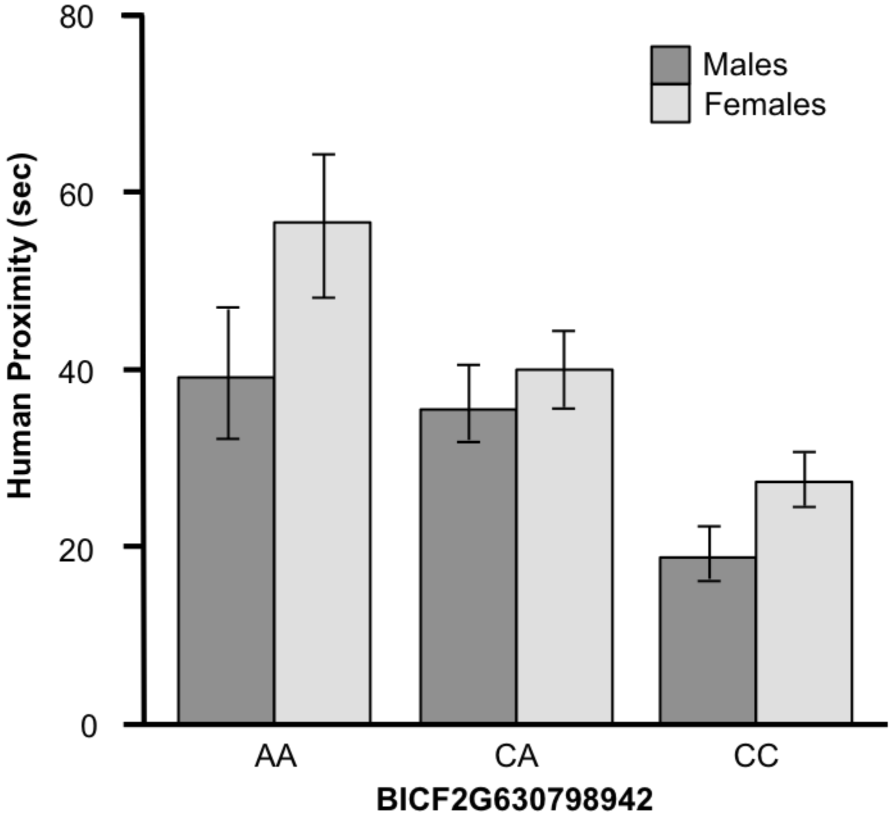

Figure S3: Histogram showing the mean of the behavior "human proximity" of each genotype of the BICF2G630798942 SNP in the complete sample of 190 + 69 dogs, separated between males and females. Both genotype (Univariate GLM: $F_{2, 253}$ = 10.961, p < 0.000) and sex (Univariate GLM: $F_{1, 253}$ = 4.920, p = 0.03) had significant effects, R^2 = 0.11. Error bars display +/- 1 SE.

BICF2S23712115/ BICF2S23712114 genotype effects on Physical Contact

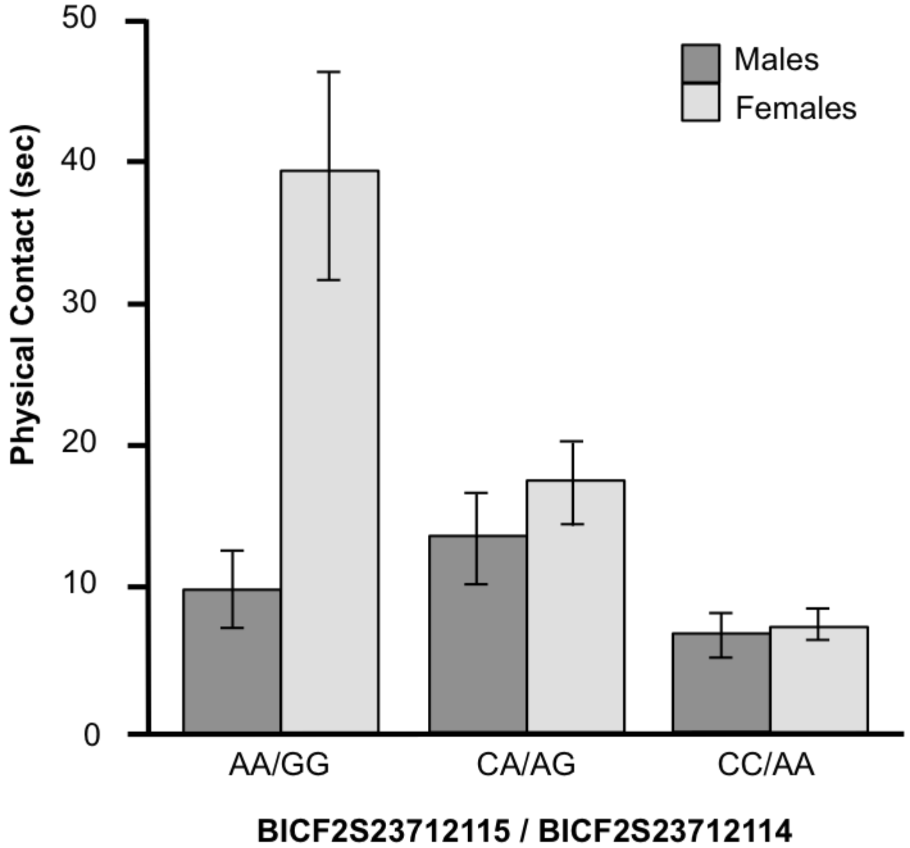

Figure S4: Histogram showing the mean of the behavior "physical contact" of each genotype of the BICF2S23712115 and BICF2S23712114 SNPs in the complete sample of 190 + 70 dogs, separated between males and females. Both genotype (Univariate GLM: $F_{3, 251} = 7.135$, $p < 0.000$) and sex (Univariate GLM: $F_{1, 251} = 4.280$, $p = 0.04$) had significant effects, $R^2 = 0.16$. Error bars display +/- 1 SE.

BICF2G630798942 genotype effects on Physical Contact

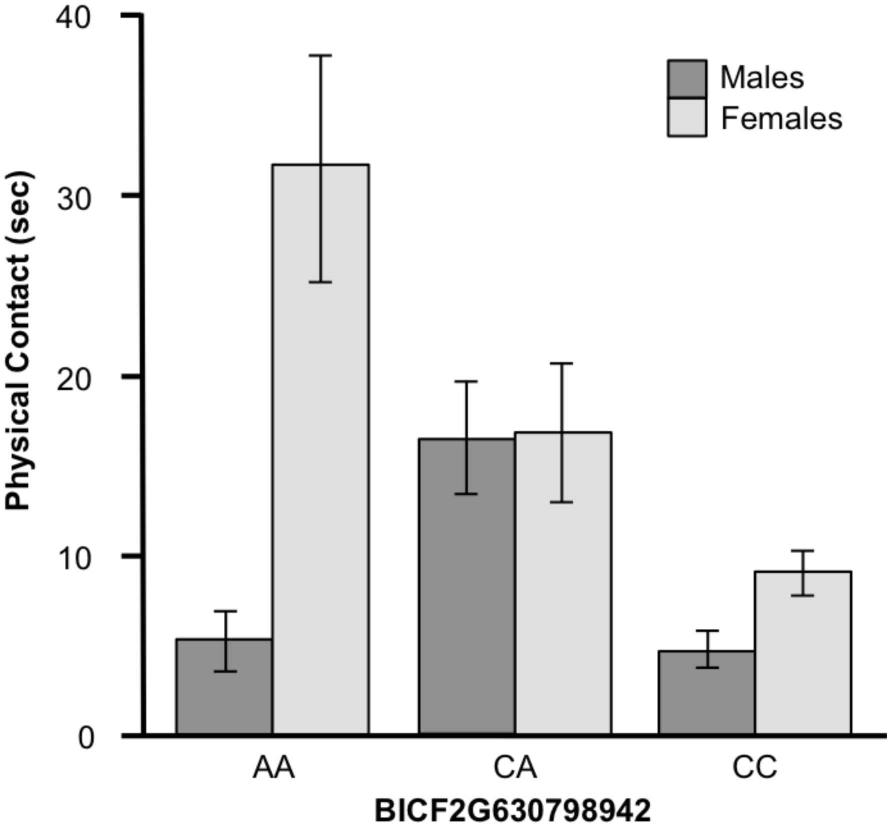

Figure S5: Histogram showing the mean of the behavior "physical contact" of each genotype of the BICF2G630798942 SNP in the complete sample of 190 + 69 dogs, separated between males and females. Both genotype (Univariate GLM: $F_{2, 253}$ = 8.246, p < 0.000) and sex (Univariate GLM: $F_{1, 253}$ = 13.541, p = 0.000) had significant effects, R^2 = 0.15. Error bars display +/- 1 SE.

BICF2G630798942 genotype effects on Human Proximity (intermediate phenotype)

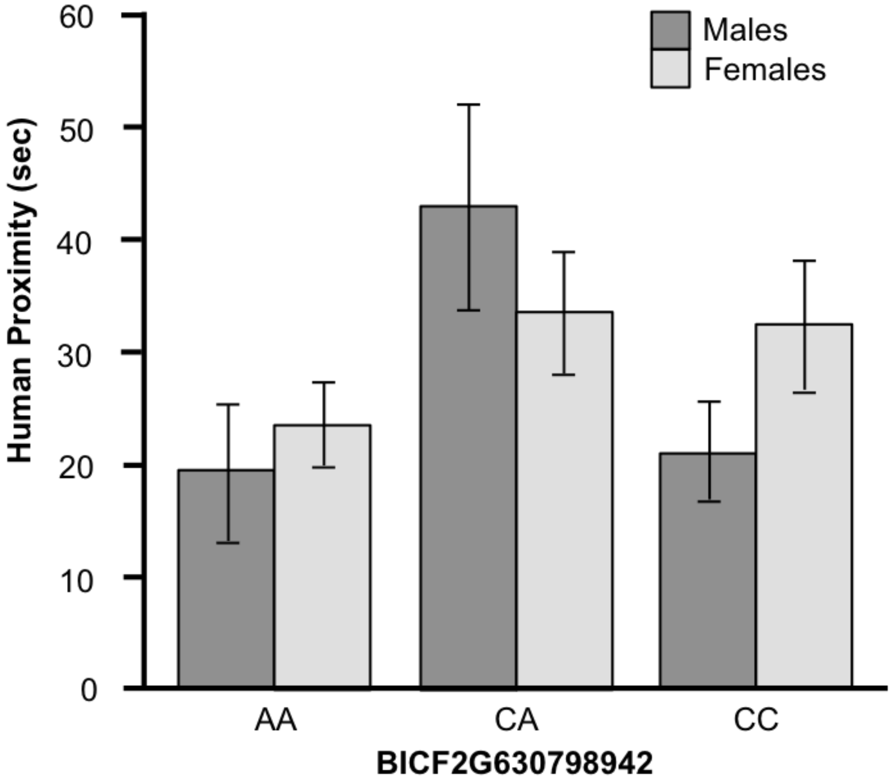

Figure S6: Histogram showing the mean of the behavior "human proximity" of each genotype of the BICF2G630798942 SNP in the additional 69 dogs with intermediate phenotypes, separated between males and females. There is no significant effect of either sex or genotype in this sample. Error bars display +/- 1 SE.

BICF2S23712115/ BICF2S23712114 genotype effects on Physical Contact (intermediate phenotype)

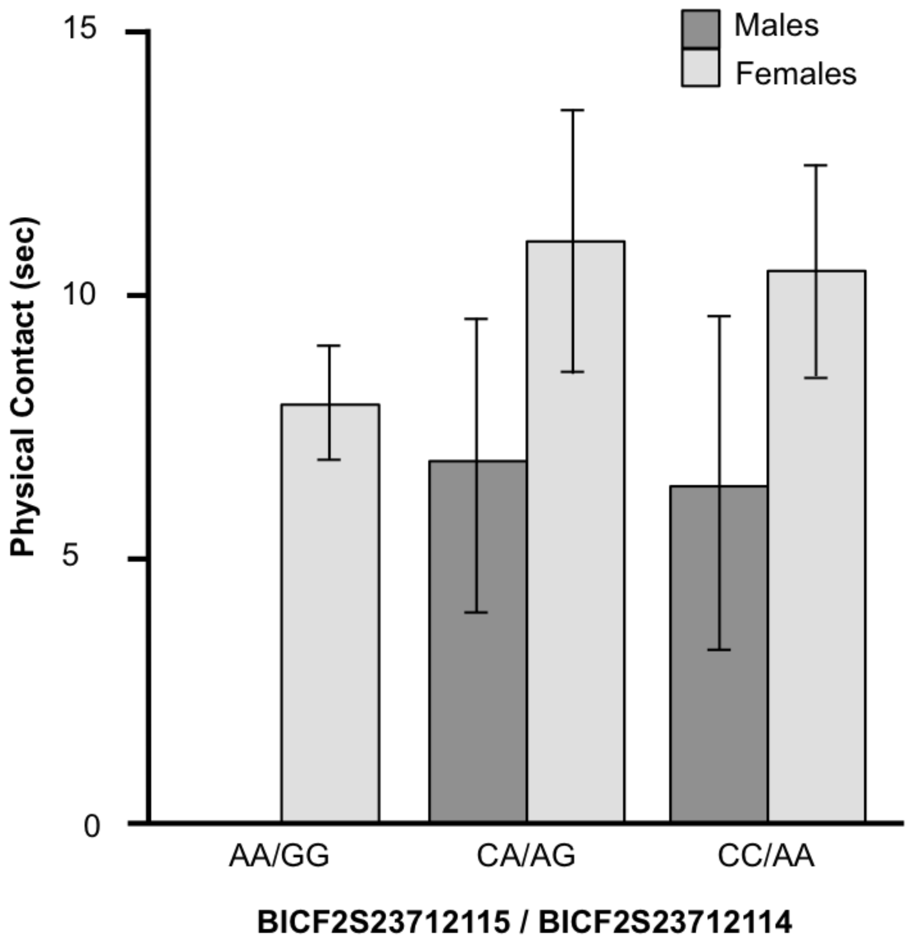

Figure S7: Histogram showing the mean of the behavior "physical contact" of each genotype of the BICF2S23712115 and BICF2S23712114 SNPs in the additional 70 dogs with intermediate phenotypes, separated between males and females. There is no significant effect of either sex or genotype in this sample. Error bars display +/- 1 SE.

BICF2G630798942 genotype effects on Physical Contact (intermediate phenotypes)

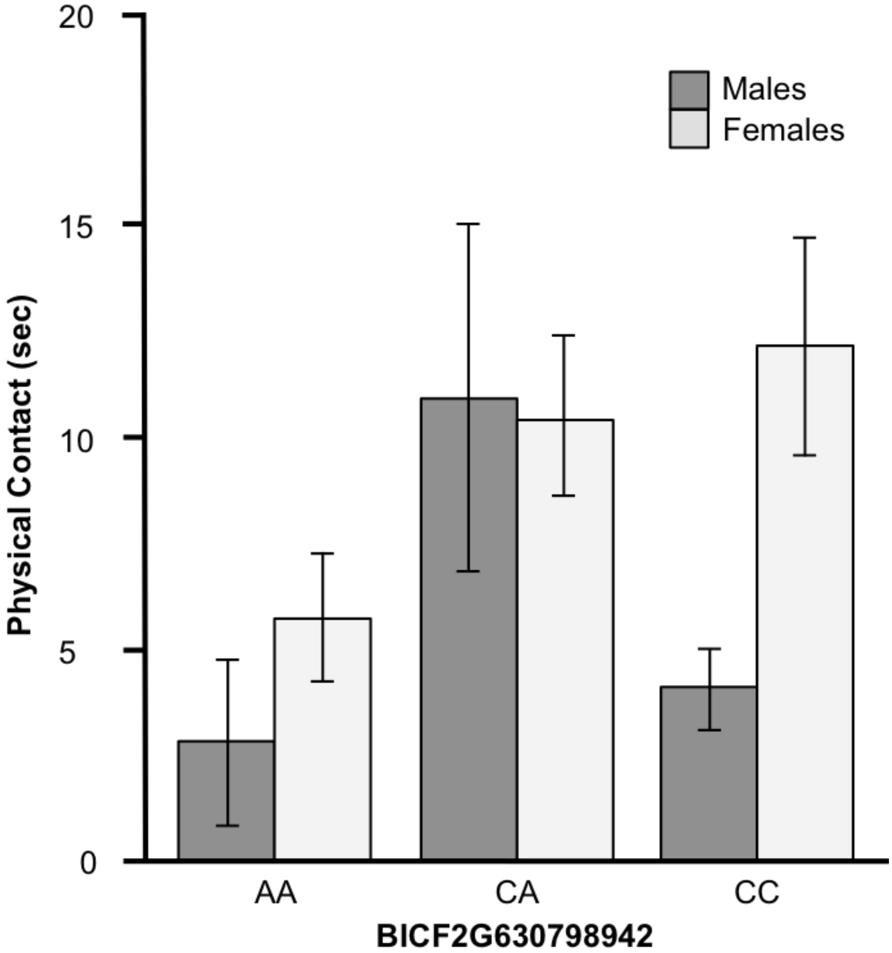

Figure S8: Histogram showing the mean of the behavior "physical contact" of each genotype of the BICF2G630798942 SNP in the additional 69 dogs with intermediate phenotypes, separated between males and females. There is no significant effect of either sex or genotype in this sample. Error bars display +/- 1 SE.

Supplementary Appendix S12

Q-Q plots of GEMMA GWAS p-values

S12 Figure 1: Q-Q plot of Duration Human Proximity GEMMA GWAS p-values with sex as a covariate.

S12 Figure 2: Q-Q plot of Latency Human Proximity GEMMA GWAS p-values with no covariate.

S12 Figure 3: Q-Q plot of Frequency Human Proximity GEMMA GWAS p-values with sex as a covariate.

S12 Figure 4: Q-Q plot of Duration Physical Contact GEMMA GWAS p-values with sex as a covariate.

S12 Figure 5: Q-Q plot of Latency Physical Contact GEMMA GWAS p-values with sex as a covariate.

S12 Figure 6: Q-Q plot of Frequency Physical Contact GEMMA GWAS p-values with sex as a covariate.

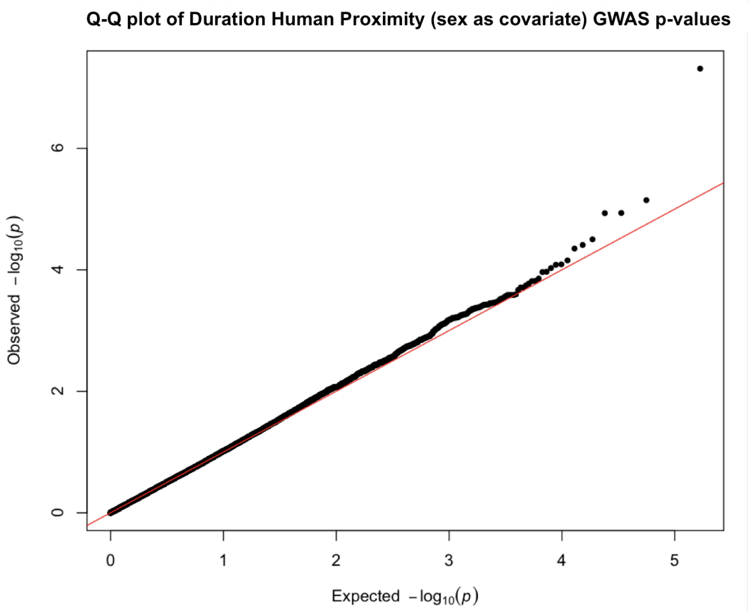

Figure 1

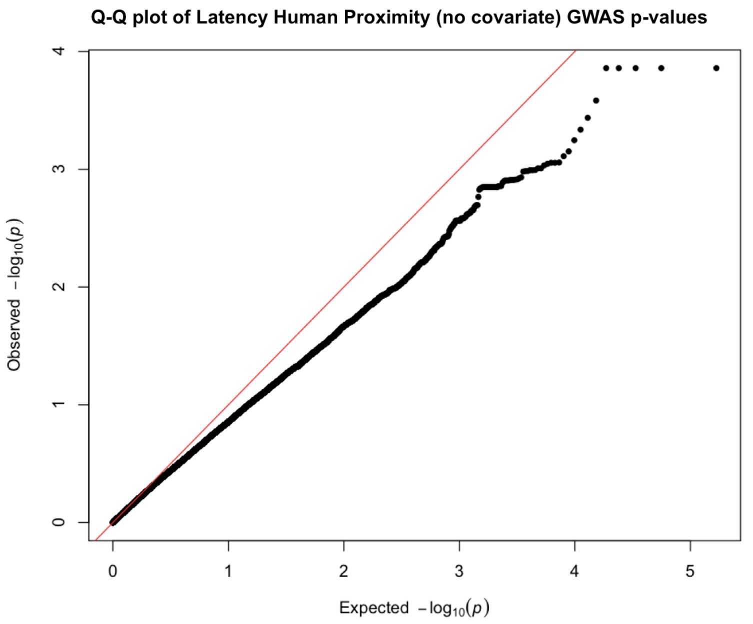

Figure 2

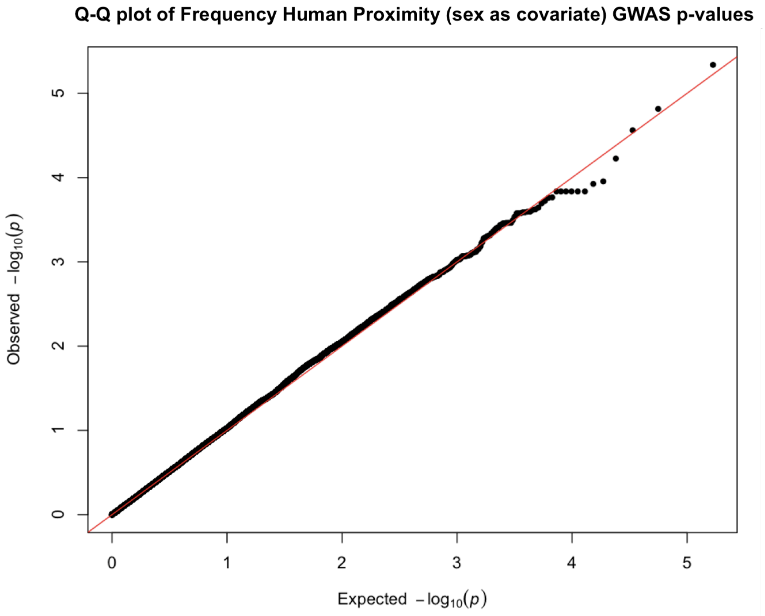

Figure 3

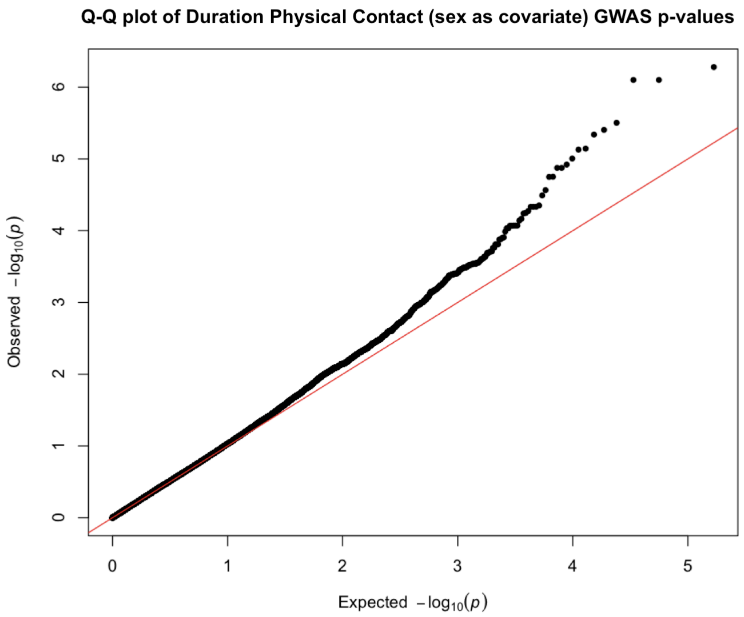

Figure 4

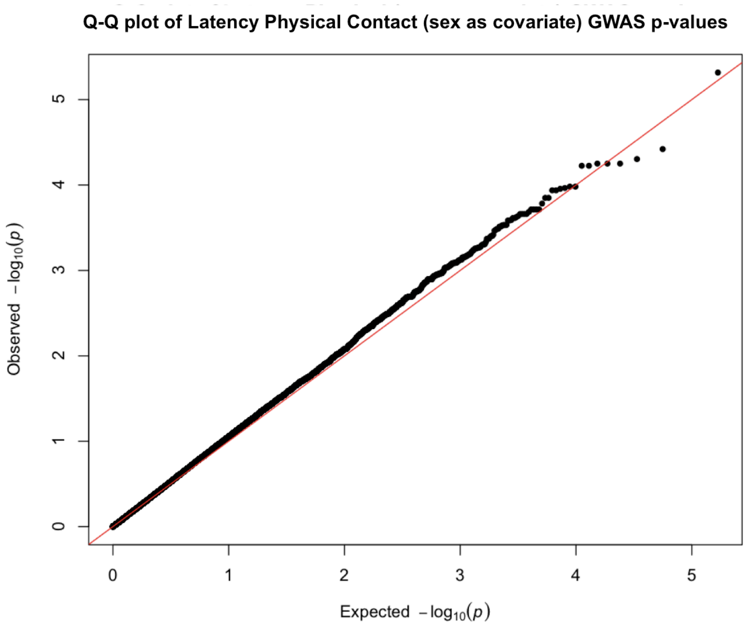

Figure 5

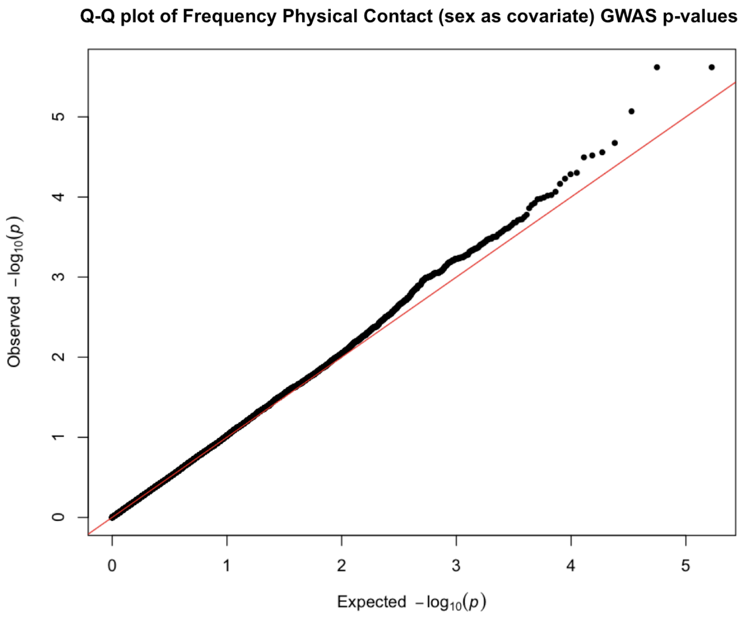
Q-Q plot of Frequency Physical Contact (sex as covariate) GWAS p-values
Observed $-\log_{10}(p)$
Expected $-\log_{10}(p)$
0
1
2
3
4
5
0
1
2
3
4
5

Figure 6

Supplementary Figure S13

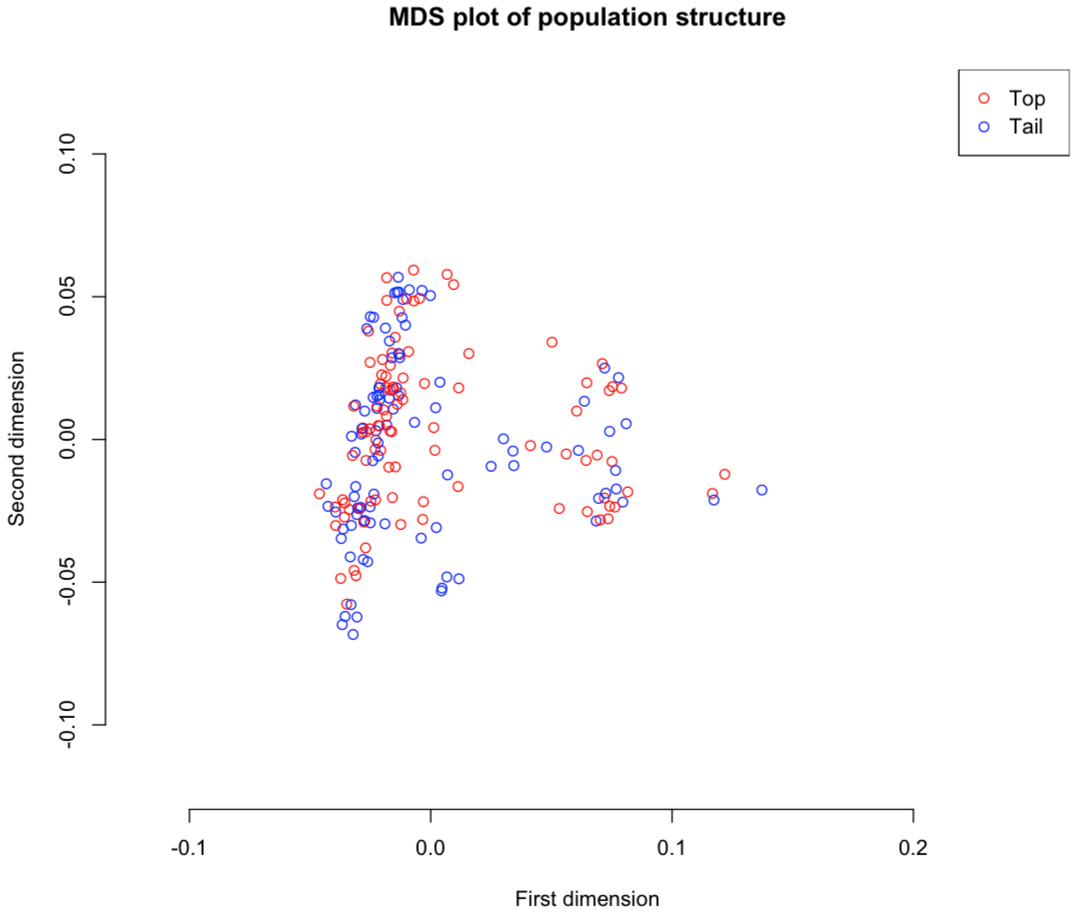

Figure S13: Multidimensional scaling plot of the first and second dimensions from MDS analysis of genome-wide IBS pairwise distances of the 190 beagles included in the GWAS. The figure visualizes the population structure with top (red) and tail (blue) dogs indicated.
