# Supplementary material for: Genomic Regions Associated With Interspecies Communication in Dogs Contain Genes Related to Human Social Disorders
Source: Sci Rep. 2016 Sep 29;6:33439. doi: 10.1038/srep33439 (PMC5041581; doi:10.1038/srep33439)
Supplement: Supplementary Table S2 [file srep33439-s2.pdf]

Supplementary Table S2

Genes associated with interspecies communication in dogs are also related to human social disorders

Mia E. Persson, Dominic Wright, Lina S.V. Roth, Petros Batakis and Per Jensen

Supplementary Table S2: Allele frequencies and phenotype means of significant and suggestive SNPs.

SNP	Chr	BP	Alleles (A1/A2)	Frequencies (%)			Means ± SE			Gene	Position
				A1/A1	A1/A2	A2/A2	A1/A1	A1/A2	A2/A2		
**Human Proximity**											
BICF2G6 30798942	26	20025266	C/A	0.43	0.39	0.18	59.69 ± 5.50	38.4 ± 3.73	22.01 ± 3.54	SEZ6L	intron 9/16
**Physical Contact**											
BICF2G6 30798942	26	20025266	C/A	0.43	0.39	0.18	6.28 ± 2.46	19.23 ± 2.59	29.23 ± 3.83	SEZ6L	intron 9/16
BICF2S2 3712115	26	29319347	G/A	0.40	0.46	0.14	6.47 ± 2.53	18.07 ± 2.38	33.15 ± 4.36	ARVCF	Intron 1/18
BICF2S2 3712114	26	29319675	A/G	0.40	0.46	0.14	6.47 ± 2.53	18.07 ± 2.38	33.15 ± 4.36	ARVCF	Intron 1/18
